# Dentistry as a Specialty of Medicine

**Published:** 1902-04-15

**Authors:** O. N. Heise

**Affiliations:** Cincinnati


### DENTISTRY AS A SPECIALTY OF MEDICINE.

BY DR. O. N. HEISE, M.D., D.D.S., CINCINNATI.

Read before the Cincinnati Odontological Society.

This subject is one which has been agitated not only by the dental profession but by medical men as well. Some months ago a symposium on dental education was held at the meeting of the American Medical Association, and I can not do better than to quote from an editorial in the *Journal of the American Medical Association*, for June 16, 1900, on this subject. "Medical educators will be deeply interested in the symposium on dental education held by the Section on Stomatology at the Atlantic City meeting of the Association. This symposium deals with every phase of the subject. The paper by Dr. N. S. Davis has special interest, and that the views therein, which were advanced more than four decades ago, are of more value to-day than when first expressed is shown in the latter discussions of the same topics by others. On perusal of these papers it will be generally admitted that advanced education is necessary for the successful practitioner of stomatology at the present time. This idea is gaining ground among dentists, and the term for their science is an evidence of the advanced conception held to-day by many dental societies of the status of dentistry as a specialty of medicine. As shown by this term, the dentist has ceased to be a mere tooth-carpenter and has become a medical scientist. Dental science has brought diseases of the mouth, jaws and teeth so obviously under the domain of general pathology that somatic problems elsewhere presented are best and most easily studied in the mouth.

This is particuliarly true of teratology, embryology, excessive and arrested development, especially as related to race advancement and degeneracy, as has been shown by Talbot. Factors of acquired degeneracy, like the drug habit, mercurialization, etc., neurotrophic and diathetic states, have nearly all their pathologic phases outlined in the interstitial gingivitis. (Talbot.) To enable the dental student to successfully treat these lesions he should have a broad training in the fundamental branches of medicine, such as anatomy, physiology, pathology, bacteriology, materia medica and chemistry. It will not do to have these sciences taught in the dental school, even by the same teachers, because a student acquires the idea that they are not necessary to a successful practitioner of dentistry. Dentistry as practiced to-day by the better element is a part of general medicine. The same requirements should be exacted for the entrance of the dental student as for the medical. They should be taught in the same class and in the same manner. The first examination should be as exacting for one as for the other, and no distinction should be made between them."

Not long ago Dr. C. M. Wright read an excellent paper on "Outline and Detail Medical Study" which pleased me greatly, as I thought he had always opposed the idea of dentistry broadening out and becoming a pure specialty of medicine, which it must do in order to fulfill his prediction that "the prescription pad and systemic remedies will be as potent weapons in the hands of the dentist in the future as are digital dexterity and instruments to-day." I might mention in passing, that Dr. F. A. Hunter, who for years has been opposed to this idea of broadening dentistry, now admits that it is only a question of time when all dentists will be medical graduates. He thinks it will not be in the near future, however, and I do not believe it to be close at hand, because the college element of our profession will hinder its rapid development, partly on account of the financial conditions involved as affecting those in control of the colleges, especially the independent ones, and also for the reason that you can not change those opinions and ideas for which men have worked for many years. It is the nature of man to cling to the thoughts handed down to him—"That which one age tells to another seems to men truth fundamental." The thraldom of the age to tradition is peculiar, but we have at the present time outgrown it to a large extent. Think, however, of the time when William Harvey discovered the circulation of the blood. He was accounted crazy, his practice declined, and a pack of "barking dogs," as he called them, was soon at his heels. "Would you have us believe that you know something that Aristotle did not know?" demanded one adversary, Dr. Primrose, adding, "Aristotle observed everything, and no one should dare to contradict him." The voice of Primrose was the voice of the age. No man over forty accepted Harvey's ideas, and half a century later the medical faculty at Paris petitioned the king to prohibit the teaching of the circulation, as a doctrine contrary to the authority of Aristotle, The same treatment has been accorded every new idea or invention. When railroads were first constructed the doctors of Bavaria declared that they would cause the greatest deterioration in the health of the people, because such rapid movement would cause brain trouble among travelers and vertigo among those who looked at moving trains.

That the dentist should have a thorough medical training is in my mind a settled fact, and it seems to me that it can not be obtained in the average dental college. Why should not the student acquire his knowledge of medicine at the fountain head, from men who have studied these branches more thoroughly than any dentist would or has the opportunity to do? Where also he can have the advantage of practical laboratory courses of instruction in all the various branches, which the dental college does not give. Again, the hospital instruction and clinical work are of paramount importance in giving broader ideas than he can get from spending all his time in the dental clinic. The chair of oral surgery gives the dental student an opportunity to witness operations other than the strictly dental ones, but he should see more than a few such operations during the year. He should obtain a knowledge of general medicine, not from books alone but by clinical instruction as well.

One of the foremost rhinologists and laryngologists in this country recently said, "The demonstration of the important relation of general medicine to dentistry, which is being shown by a number of scientific articles, is a neglected field. The relation of facial contour and the upper arch to nasal breathing in early childhood can not be overestimated, in fact, it is nasal breathing which controls the regular development of the facial bones and especially the superior maxilla. A thin alveolar process in the upper jaw, from lesions of the teeth, may cause by extension ot inflammation, by continuity of structure, lesions of the floor of the nose or of the antrum; or, on the other hand, deflections of the septum or spurs situated close to the floor of the nose, by the inflammatory action set up on the surrounding structure, may bring about inflammation and diseased condition of the teeth in direct line of obstruction. The stomatologist should have a thorough knowledge, not only of the nasal cavities and accessory sinuses, but also of general medicine, and, indeed, a general practitioner or specialist should have a more thorough knowledge of stomatology."

The administration of anesthetics, the injection of cocain, etc., as ordinarily done by the dentist is likely to bring discredit upon the profession. Patients are given nitrous oxid gas when the dentist has no idea of their general condition. In fact, is the average dentist equipped to ascertain even in a general way the condition of the heart, lungs, kidneys, etc., and in case of any serious trouble during the administration is he qualified to take care of the patient? Does he not invariably call in a physician instead of a brother dentist, thereby acknowledging that we, as dentists, are not competent to undertake cases where life is at stake. What would be the criticism in a fatal case where no physician was called? Would it be the same if the accident or fatality occurred at the hands of an oculist, aurist, rhinologist, laryngologist, or any other medical specialist, who had not called in medical aid? We know that the dentist would be censured, in fact, it has even been suggested in certain quarters that dentists should be prohibited from giving anesthetics except in the presence of a physician. Other countries, like France, will not allow American dentists to administer anesthetics, or even inject cocain, etc., unless they have complied with the French law regarding dental education, as over there they require a better medical training than our dental colleges give. While I was in Paris last summer this was told me by an Ann Arbor dental graduate. He had intended to begin practice in Paris, but before he could do so he had to spend some two years in study and in attendance upon one of the dental colleges. At first he considered this a hardship and an injustice, as he felt capable of undertaking any dental operation as well as any French dentist, but when the ordeal was past he realized the deficiency of our method of giving only theoretical medical training in the dental schools.

Some men, like Dr. James Truman and others, advance the idea that "in proportion as we aspire to build up our curriculum so as to produce M.D.'s, just in that proportion will dentistry deteriorate." This is in line with the ideas held by the past generation of business men, that boys who had a high-school or university education did not make good business men, as such education prevented a thorough training in business, the early and adaptable years being consumed in acquiring knowledge which could not be used afterwards. The facts are. however, that the leaders in business enterprises to-day are men of broad and liberal education, and where it was not obtained in college it has been acquired by self-education in after years, showing that these men felt the need of it. It is a common thing to-day to see graduates of universities enter business houses and owing to their superior mental training, master the details of the work in a short time, where failure would have resulted without the trained mind. Those dentists who object to a medical training for the dental student do not understand nor appreciate the full scope and importance of our profession. Those men who are satisfied with filling teeth and making plates are like the simple-hearted ancient geographers who wrote on their maps of the world, besides the columns of Hercules (representing the Straits of Gibraltar), "Here ends the world."

One of the principle objections urged against this broadening of our education, or as Dr. Wright puts it,

"The bringing up of our outline work," is that the dentist thereby loses in fingercraft or dexterity of manipulation, that he becomes too theoretical and not practical enough. I fail to see why he should become less expert on account of being better equipped mentally, for in the very act of obtaining the desired knowledge he is required to do considerable work with his fingers, and the laboratory courses demand much manipulative ability. We see the result of such study in the men who take up the various specialties of medicine. Look at the oculist, aurist, the general and special surgeon, in their various fields of work—are they any less expert in their operations or handling of instruments than is the dentist? Many of them will outstrip him in skill and dexterity. Consequently, it seems to me that a thorough scientific medical training does not detract one iota from a dentist's manual dexterity.

### DISCUSSION.

Dr. C. M. Wright: When we treat an exposed pulp, an abscess, a case of pyorrhea, or any lesion of the parts closely related to the teeth, we are practicing a specialty of medicine in as strict a sense as does any other specialist. So much must be conceded, whether we accept or reject the dictum that dentistry is a specialty of medicine. During the last thirty years a decided change has taken place in dental practice. Who at that time treated pyorrhea? The majority of dentists then extracted all so-called ulcerated teeth. When a few thoughtful, progressive men began to treat these affections they were unconsciously but none the less certainly taking the first steps toward establishing dentistry as a specialty of medicine. If we have any real ambition to be recognized as part of the great medical body we can attribute our failure to have already attained that end to our custom of basing our estimate of each other on the ability to fill teeth, treat abscesses, make dentures, etc., and to the common fault of openly detracting from one another's skill as a dentist by such statements as "He is a poor operator and plate-worker," etc, I think we are not specialists in medicine because the principles of practice in medicine and in dentistry are radically different. While I in a way agree with Dr. Heise that dentists should have a more liberal medical education, and trust the profession will come up to the standard he sets, I say the greatest drawback to the realization of such a hope is in the present failure of the rank and file to recognize the necessity of the step. If any one of us should give up the filling of teeth and everything that pertains to the purely mechanical nature of our services, and devote himself solely to treating oral lesions, he would then become a medical specialist within our own lines.

Dr. F. A. Hunter: I do not believe the dentist is any better as a dentist for having the medical degree, and the leaders of our profession to-day are those who do not hold it. At present we are a separate and distinct profession, having our own colleges, literature, methods and aims in the nature and substance of what is taught the students. The time may come when the medical degree will be required of the dental matriculate, but I doubt whether that time is near at hand. I furthermore question the correctness of Dr. Heise's assumption that anatomy, physiology, therapeutics and allied subjects are any more readily assimilated by the dental student in a medical college than by the same student under the teachings of a medical professor in a dental college. Why should they be? Of course it is obvious that the dentist often verges on the borderland of medicine and sometimes actually practices it, but for all that the practice of dentistry is so essentially different in most respects from that of medicine that they may be said to have very little in common. The relationship of the two is not of sufficient importance to make it incumbent upon the dentist to qualify himself as a physician. I have said that generally we as dentists are not regarded as medical specialists. In hiring the dentist under contract the United States government recognizes him merely as a laborer, and he has no rank as an officer as has the army surgeon.

Dr. W. M. Williams: I thoroughly approve of the advanced position Dr. Heise takes in his paper, and I have always advocated raising the standard of dentistry as high as possible, on the principle that what we claim for ourselves the public will ultimately accord us. Dr. Hunter puts the matter in an unnecessarily harsh light in using the word "laborer." In both the army and navy the government employs surgeons and physicians under the same conditions of contract, and no one regards it as a reflection on the medical profession. To be sure, the army and navy have also other surgeons who hold the regular title and rank of officers, to which the dentist has no chance of attaining, but the regular officers have always opposed the conferring of any army or navy titles on surgeons or physicians, so the dentist is no more humiliated than the surgeon has always been. Furthermore, the dentist has only recently been admitted to either branch of the service, and we have sufficient ground for congratulation that so much has been achieved, without endeavoring to detract from the honor of the recognition which our profession has received from the government.

DR. H. T. SMITH: I do not accept Dr. Heise's statement that throat, nose, eye, ear and other specialists have digital dexterity and skill equal or superior to that of the trained dentist, for the average dental operator would easily take precedence over the average operator in any medical specialty. It has been asked whether even the making of a bridge does not depend on a certain anatomical preparation of the teeth which may be characterized as belonging to medical science. This might be conceded as a matter not particularly significant in its bearing on the question. Dr. Heise assumes that the dental student is not well grounded nor ordinarily interested in pathology, histology and other branches which receive much attention in the medical school, but this is a mistake, and the dental student is as likely to be deeply interested in these subjects as is the medical matriculate. One thing which would militate against having the dental student first take the medical degree is, that it would in most cases postpone his entrance into the dental college until the twenty-fourth or twenty-fifth year, and at that age the ability to acquire finger-craft is not so great as earlier in life.

DR. J. R. CALLAHAN: In the main I heartily endorse Dr. Heise's views, and I have always regretted that I did not take a full medical course before entering the dental college. The question may be asked why dentists were not long ago appointed to the army, and I believe it is because of the estimate which they placed on themselves and because of the opinion of the dental profession which they have caused to go throughout the world. Another thing which has exerted its influence against us is the fact that a large number of dentists have received the honorary "M.D." title, but very few have actually taken the hospital course.

Dr. F. W. Sage: I am distinctly in favor of anything which makes for the better equipment of the dentist and the elevation of the profession, but I must still maintain that dentistry is *sui generis*, and that it can not be merged into the medical profession because it ministers to its patients in a wholly different way, which is largely mechanical. The question is not entirely what we would like to be, but what our patients wish us to be. I will go further than Dr. Hunter and say that some of the most brilliant men in the history of the dental profession were incapable of profiting by a medical education. They were so distinctly mechanical in the quality of genius they displayed that they could never become interested in the generality of the subjects which engages the medical student. Nevertheless, they served their patients well and were esteemed peerless as dentists. At the present day men with meager attainments in medical knowledge are serving their patients with the same degree of satisfaction. No argument is required to establish the fact that when the average of young men who apply for matriculation at the dental colleges is taken, a superficial observation shows that they have not the mental make-up of medical students. They are moved to enter a dental college largely by mechanical instincts. A man w.th this mechanical bent can not be forced to absorb a large amount of theoretical knowledge on subjects not closely allied to the mechanical subject of dentistry. The question is one of temperament, and it is vain to attempt to force upon any man a system of training which meets no response in the impulses of his nature.

As regards the medical profession, it is a question whether the men who have given dentistry the most encouragement to fully identify itself with the parent body have the slightest appreciation of the patient application necessary to acquire the digital facility which alone renders the dentist of special value to his patients. The physician admires the dentist who comes nearest to being a physician, for then for the first time he understands him, but he does not at all understand him as a dentist and does not especially admire him. Why should the dentist expect to practice even plastic surgery of the face or mouth? What are specialists for? Why does not the rhinologist or laryngologist occasionally operate on the teeth? Granting that a large majority of the dentists could be qualified to practice oral surgery in the fullest significance of the term, would they be called on to any considerable extent to so practice, and could they be expected to compete successfully with the surgeon enjoying a far greater range of opportunity in the same line? As a matter of fact, dentists who practice dentistry acceptably usually have their hands full. To keep pace with the advancement of strictly dental science is enough for the average man. Finally, all the knowledge of medicine which the dentist needs he should be able to get in the dental college. For what other purposes have the colleges extended their course a year, looking to a further extension in the near future? On what rational ground can it be maintained that chemistry, therapeutics, anatomy, histology, etc., can not be as thoroughly and satisfactorily taught in the dental college as in the medical college? Personally, I freely admit my regret that I did not take a full medical course, but at the same time I am not in sympathy with anything which belittles dentistry as regards its standing independent of any closer relationship to the medical profession, and I believe the dental colleges are in all respects the best medium for qualifying the incoming dentist for the practice of his profession.

Dr. H. A. Smith: The trend of modern education is to fit the young man specially for the occupation which he is to follow. Beyond that there can be no objection whatever to an acquisition of broader knowledge. The requirements made upon us as dentists are gradually broadening and our colleges are extending their courses of study to meet these demands. Our essayist was a skillful dentist when he held only the dental degree, but having acquired the degree of M.D. for the purpose of practicing rhinology in connection with dentistry, he naturally thinks he acquired useful knowledge for the practice of dentistry, that he would not otherwise possess. I contend that Dr. Heise, with his habits of study, would have acquired the needful knowledge without the aid of strictly medical teachers.

Dr. Heise, closing discussion: I can not agree with the opinion repeatedly expressed here to-night, that the dental colleges afford the same advantages to the student in histology, pathology, bacteriology and physiology as do the medical schools, for the former lack the advantages of properly conducted laboratory courses and the practical clinical instruction in medicine. Of what use is book knowledge of medicine without practical application of same? In this regard the dental colleges will always be deficient.

As to the statement that dentistry is *sui generis* and can not be merged into the medical profession because of its mechanical nature, I would reply that it is not more so than some of the specialties of medicine. Ophthalmology is perhaps more like dentistry than any other in this respect, being largely mechanical. Take, for instance, the examination of the eye and the prescribing of glasses—a mechanical operation in every sense of the word. It is true that the oculist does not grind his own lenses, make his own spectacle frames, etc., but a great deal of the purely technical in dentistry could and should be much better done by men expressly trained for the purpose, working under the supervision of the dentist.

As to the statement that the average dentist possesses more digital dexterity and skill than the average medical specialist, we must not imagine that other specialists are bunglers in this respect, for many of them are first-class operators and equal to any dentist. Some of them might be better, but the majority of dentists are not up to the standard.

It is to the disadvantage of dentistry that it was ever divorced from the medical profession, as happened in the time of Dr. Chapin A. Harris, for it would have been further advanced as a science and have been more efficient in many ways than it is to-day if that separation had never taken place.—*Digest*.

